# Is Insomnia Linked to Sleep Bruxism in Adults? A Systematic Review and Meta‐Analysis

**DOI:** 10.1111/joor.70068

**Published:** 2025-10-04

**Authors:** Eduardo Machado, Jessica Klöckner Knorst, Fabrício Batistin Zanatta, Thais Perez Iglesias, Cibele Dal Fabbro, Dalva Poyares

**Affiliations:** ^1^ Sleep Medicine and Biology Division, Department of Psychobiology Universidade Federal de Sao Paulo Sao Paulo Brazil; ^2^ Postgraduate Program in Dental Sciences Universidade Federal de Santa Maria Santa Maria Brazil; ^3^ Faculté de Médecine Dentaire Université de Montréal Montreal Quebec Canada; ^4^ Centre de Recherche Institut Universitaire de Gériatrie de Montréal (CRIUGM) Montreal Quebec Canada

**Keywords:** adults, insomnia, oral health, sleep bruxism, systematic review

## Abstract

**Aim:**

Systematically evaluate the previous literature on the association between insomnia and sleep bruxism (SB) in adults.

**Methods:**

Advanced searches were performed in different databases (PubMed, Embase, LILACS, Scopus and Web of Science) and grey literature until March 2025. Two trained reviewers independently conducted all stages of the review to identify observational studies evaluating the association between insomnia and SB in adults. Methodological quality was assessed using the Newcastle–Ottawa Scale (NOS). A narrative synthesis summarised the main characteristics of the included studies. Meta‐analyses were performed to obtain pooled estimates separately for self‐reported and polysomnography (PSG)‐based SB. Available data on insomnia and SB were converted into odds ratio (OR) with corresponding 95% confidence intervals (CIs).

**Results:**

Of the 1135 records initially identified, 931 were screened by title and abstract, and 23 were assessed in full text. Eight studies met the inclusion criteria for the systematic review, and six were eligible for meta‐analysis, comprising a total sample of approximately 6990 adults. The meta‐analysis of four studies investigating the association between insomnia and self‐reported SB found no statistically significant association under the random‐effects model (OR 1.17; 95% CI 0.79–1.72). Likewise, the pooled analysis of studies assessing PSG‐based SB also showed no significant association with insomnia (OR 0.91; 95% CI 0.43–1.95).

**Conclusion:**

Our findings indicate a lack of consistent evidence for a significant association between insomnia and SB. This conclusion is further limited by the small number of included studies, the moderate risk of bias in some studies, and the observed heterogeneity.

## Graphical abstract

This systematic review and meta‐analysis evaluated the association between insomnia and sleep bruxism in adults. The findings showed no consistent evidence of a significant relationship between the two conditions. Further high‐quality studies are needed to clarify this potential association.

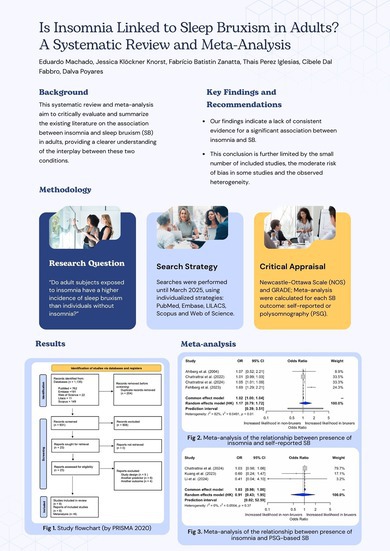

## 1 Introduction

Insomnia is a common sleep disorder characterised by difficulties initiating or maintaining sleep, early morning awakenings, or non‐restorative sleep, accompanied by significant daytime impairment [1]. The condition can be acute or chronic, with chronic insomnia typically defined as symptoms occurring at least three times per week for a minimum of 3 months. Diagnosis is clinical and based on standardised criteria, such as those outlined in the International Classification of Sleep Disorders (ICSD)—3rd ed. and 3rd ed. revised (ICSD‐3) [1, 2] and the Diagnostic and Statistical Manual of Mental Disorders—Fifth Edition (DSM‐5) [1, 2, 3]. Recent guidelines, including the 2024 Brazilian Consensus on Insomnia, emphasise a multidimensional assessment that integrates subjective complaints, sleep patterns, and the impact on functioning [4].

The global prevalence of insomnia symptoms is estimated to range between 10% and 30%, and it has been associated with a variety of adverse health outcomes, including increased risk for cardiovascular disease, mood disorders, and impaired quality of life [5, 6]. Moreover, insomnia has been linked to altered physiological arousal and sleep fragmentation, which may play a role in the development or exacerbation of other sleep‐related conditions. Thus, sleep bruxism (SB) is defined as a masticatory muscle activity during sleep characterised as rhythmic (phasic) or nonrhythmic (tonic) activity, and it is not a disorder in the absence of complications or comorbidities [7, 8] – has emerged as a potential association with sleep disorders.

SB aetiology is multifactorial, involving central and autonomic nervous system mechanisms, genetic predisposition, and psychosocial and behavioural factors [9, 10, 11]. Still, evidence showed that SB may be associated with neurodegenerative [12] and inflammatory processes [12, 13]. Importantly, the presence of insomnia symptoms has been hypothesised to contribute to the occurrence of SB through increased cortical and autonomic arousal during sleep [10, 14]. However, the strength and directionality of this association remain unclear, and findings across studies are heterogeneous—partly due to differences in study design, diagnostic criteria, and methods of SB assessment [9, 15].

One of the first studies to show an association between SB and insomnia was the one from Maluly et al. [15], and after that another publication, based on the same population, showed that different phenotypes may explain this association in some groups of patients [16]. Of particular relevance is comorbid insomnia and obstructive sleep apnea (COMISA), a condition with substantial health implications that requires careful differentiation from OSA alone. In a recent study, the bruxism episode index (BEI) and other SB parameters were not found to be statistically different between the OSA and COMISA groups [17].

Although several individual studies have examined the potential relationship between insomnia and SB, to our knowledge, no systematic review has yet synthesised the available evidence on this topic. Given the growing recognition of insomnia as a modifiable risk factor for sleep‐related disorders and the clinical implications of SB for oral health and quality of life, a comprehensive synthesis of current findings is needed. This systematic review and meta‐analysis aim to critically evaluate and summarise the existing literature on the association between insomnia and sleep bruxism in adults, providing a clearer understanding of the interplay between these two conditions.

## 2 Methods

This study was developed in accordance with the Preferred Reporting Items for Systematic Review and Meta‐Analysis (PRISMA) protocol statement (Table S1). It was prepared and registered in the International Prospective Register of Systematic Reviews (PROSPERO) database under registration number CRD420250653542.

### 2.1 Research Question and Eligibility Criteria

The following focused question was structured: “Do adult subjects exposed to insomnia have a higher incidence of sleep bruxism than individuals without insomnia?” Studies that answer the research question and include the following population, exposure, comparison, and outcome (PECOS) were included: Population (P) = adult individuals (≥ 18 years) of both sexes; Exposure (E) = insomnia diagnosed by validated index [1, 3, 18] or by reporting symptoms of insomnia (difficulty initiating sleep, difficulty maintaining sleep, or early awakening); Comparator (C) = subjects without a diagnosis of insomnia; Outcome (O) = subjects with possible, probable, or definitive SB [7] or self‐report, clinical examination, or device‐based SB [8]; Study design (S) = cross‐sectional studies with comparison group, case–control, or cohort, published in any language and without restriction on publication date.

Studies that do not present an observational design, such as intervention studies, reviews, and case series, were excluded, as well as observational studies that have evaluated specific samples, such as subjects with genetic syndromes, mental disorders, or craniofacial anomalies.

### 2.2 Search Strategies

Advanced searches were performed in different databases until March 2025, using individualised strategies: PubMed, Embase, LILACS, Scopus and Web of Science (Table S1). A combination of “MeSH terms” (Medical Subjects Headings) in “Entry” terms and similar terms was initially conducted in the PubMed database and adapted for the other databases. Grey literature was identified through Google Scholar (first 100 links) and the references of the studies included in the review.

### 2.3 Study Selection

First, all studies retrieved from the five (5) databases were compiled and duplicate records were identified for subsequent exclusion. This process was conducted in the Rayyan software (Qatar Computing Research Institute, http://rayyan.qcri.org), which also supports the article selection phase. This process was carried out in two phases. In the first phase, titles and abstracts were read to identify potentially eligible records. When there is no clarity for decision‐making due to a lack of information in the title and abstract, the study was considered potentially eligible. In the second phase, the studies were read in full to confirm or deny their eligibility. Disagreements in this phase were resolved by consensus among the reviewers. Both phases were be conducted in duplicate and independently by two previously trained reviewers (EM and JKK). Kappa evaluated the examiners' agreement on the selection of studies and was equal to 1.00.

### 2.4 Data Extraction

Data extraction was conducted by two independent reviewers (EM and JKK), with the help of an extraction form previously prepared in Excel. A third reviewer (DP) cross‐referenced the collected data to confirm its consistency. The following data were collected: (1) citation (first author and year of publication); (2) location (country); (3) study design; (4) sample characteristics (population, sample *N*, age); (5) evaluation criteria and/or definition of insomnia and sleep bruxism; (6) adjustment for confounding variables; (7) mean or frequency according to the predictor and outcome; and (8) measure of association presented (crude and adjusted—most fully adjusted model if there is more than one adjusted model).

### 2.5 Bias Risk Assessment

Two independent authors (EM and JKK) determined the risk of bias of the included studies using the Newcastle‐Ottawa scale (NOS) [19] for case–control and cohort studies and adapted for cross‐sectional studies. The selection, comparability, and outcome/follow‐up domains were assessed by eight items distributed among them. The NOS scale score can range from 0 to 9, indicating a lower risk of bias for scores closer to 9 [15]. Studies scoring 0–4 will be considered at high risk of bias, 5–6 at moderate risk, and 7–9 at low risk [20].

### 2.6 Certainty of Evidence

The certainty of evidence was assessed using the Grading of Recommendations Assessment, Development and Evaluation (GRADE) approach. This method considers five domains that may lower the certainty of evidence—risk of bias, inconsistency, indirectness, imprecision, and publication bias. For each outcome, the overall certainty was rated as high, moderate, low, or very low. Observational studies, by themselves, are already considered to have low strength of evidence. Points to be assessed such as the risk of bias, inconsistency, indirect evidence, imprecision, and publication bias may further reduce the certainty of evidence. However, other points, such as the occurrence of a large magnitude of effect, dose–response gradient, and the investigation of confounding factors may increase the certainty of evidence [21, 22].

### 2.7 Synthesis of Data and Analysis

Estimates of the association between insomnia levels and SB were pooled through meta‐analysis. Both common and random‐effects models were calculated for each SB measure: self‐reported or polysomnography (PSG). A random‐effects meta‐analysis was performed using the Hartung‐Knapp (HK) method, which provides more accurate confidence intervals, especially with few studies or substantial heterogeneity. However, the random‐effects model was selected for final interpretation due to its conservative approach in accounting for between‐study heterogeneity. Effect estimates from primary studies were converted into log‐binomial and then transformed into odds ratio (OR) with 95% confidence intervals (CI) [23, 24]. To maintain data independence, only one study per overlapping sample was included in the meta‐analysis, selected based on sample size, risk of bias, and analytical rigour. Heterogeneity was examined using the *I* ^2^ statistic. The *I* ^2^ ranges from 0% to 100%, and is classified as follows about heterogeneity: 0%–30% (not important); 30.1%–50% (moderate); 50.1%–75% (substantial); and 75.1%–100% (considerable) [25]. All statistical analyses will be calculated using RStudio software (version 4.2.2).

The assessment of publication bias using funnel plots or statistical tests (e.g., Egger's test) was not performed due to the limited number of studies included in the meta‐analyses.

## 3 Results

**FIGURE 1:**
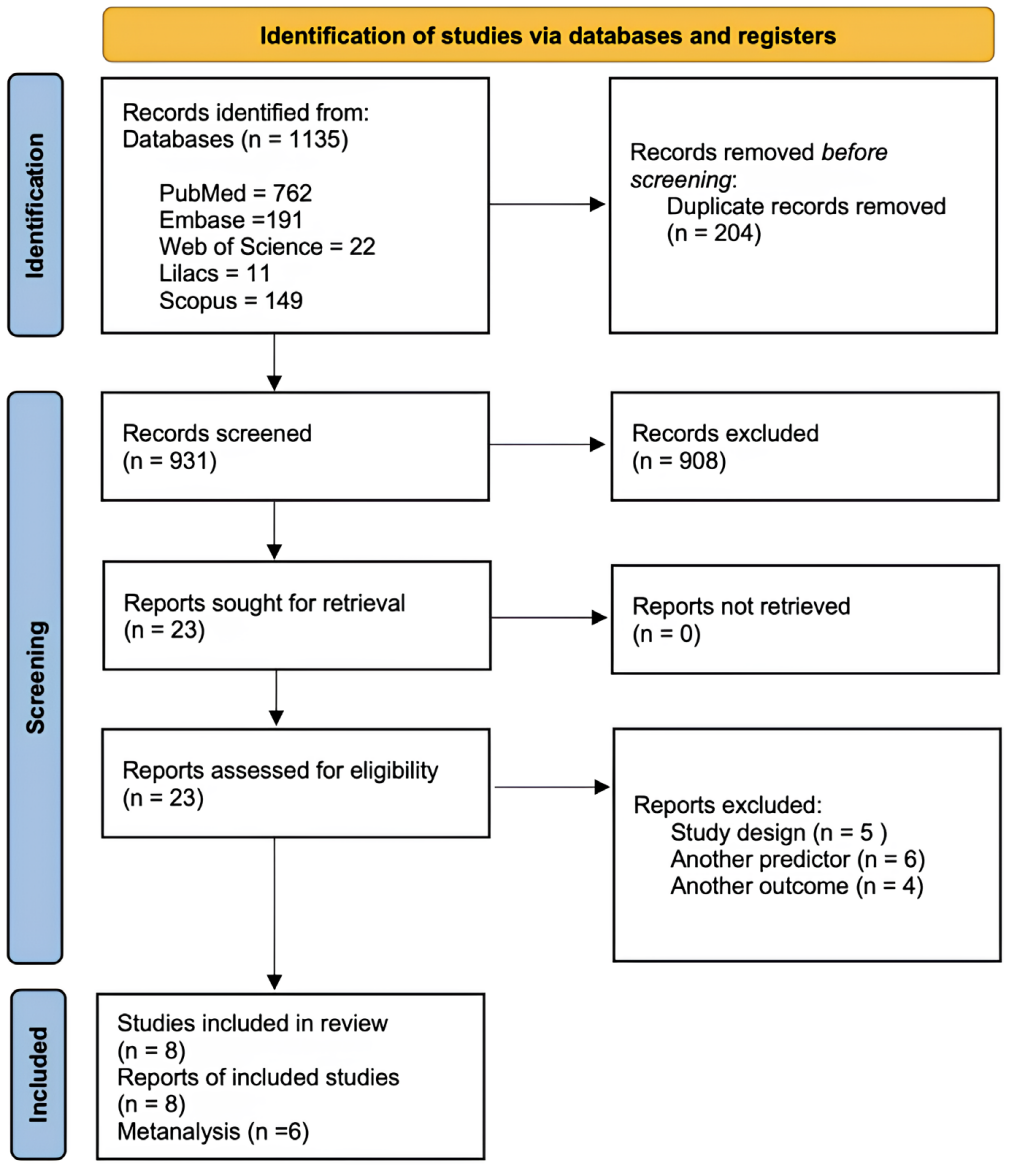
Study flowchart (by PRISMA 2020).

A systematic search across five databases initially identified 1135 records. After removing 204 duplicates, 931 articles remained for title and abstract screening. Of these, 908 were excluded based on the predefined eligibility criteria, resulting in 23 full‐text articles assessed for eligibility. Following full‐text evaluation, 15 articles were excluded due to study design (*n* = 5), inappropriate predictor (*n* = 6), or incompatible outcome measures (*n* = 4). Consequently, eight studies were included for narrative synthesis and six included in the meta‐analysis (Figure 1). After accounting for overlapping samples—specifically, three studies that analysed data from the same population [15, 16, 26]—the total number of unique participants included across the studies was approximately 6990 adults.

Among the eight studies included in the systematic review, the majority were conducted in Brazil (*n* = 4) [15, 16, 26, 27], followed by the Netherlands (*n* = 3) [28, 29, 30] and Finland (*n* = 1) [31]. Regarding study design, cross‐sectional studies predominated (*n* = 7) [15, 16, 26, 27, 28, 29, 30], while only one study adopted a cohort design [31]. The methods used to assess insomnia varied across studies. Four studies employed validated instruments such as the Insomnia Severity Index (ISI), while others applied diagnostic criteria from the DSM‐IV/V and the ICSD. Two studies relied on self‐reported measures of insomnia based on a yes/no response. In terms of SB assessment, three studies used only self‐reported measures, two employed only device‐based criteria, quantifying RMMA episodes per hour of sleep through PSG, and three used both methods. Of the included studies, 50.0% were classified as having a moderate risk of bias (Table 1).

**TABLE 1:** Narrative synthesis of the studies included in the systematic review (*n* = 8).

Author	Country	Design	Age	*N*	Insomnia	Sleep bruxism	Risk of bias
Ahlberg et al. (2004) [31]	Finland	Cohort	30–50	205	Self‐reported	Self‐reported	Moderate
Chattrattrai et al. (2022) [28]	Netherlands	Cross‐sectional	18–87	2251	ISI	Self‐reported	Low
Chattrattrai et al. (2024) [26]	Brazil	Cross‐sectional	20–79	1042	ISI	PSG (RMMA) Self‐reported	Low
Fehlberg et al. (2023) [27]	Brazil	Cross‐sectional	> 20	1986	Self‐reported	Self‐reported	Moderate
Kuang et al. (2023) [29]	Netherlands	Cross‐sectional	35–60	86	ISI, DSM‐V and ICSD‐III	PSG (RMMA)	Moderate
Li et al. (2024) [30]	Netherlands	Cross‐sectional	> 18	292	Self‐reported	PSG (RMMA)	Moderate
Maluly et al. (2013) [15]	Brazil	Cross‐sectional	20–80	1042	DSM‐IV	PSG (RMMA) Self‐reported	Low
Maluly et al. (2020) [16]	Brazil	Cross‐sectional	20–80	1042	ISI and DSM‐IV	PSG (RMMA) Self‐reported	Low

Abbreviations: DSM, Diagnostic and statistical manual of mental disorders; ICSD, International Classification of Sleep Disorders; ISI, Insomnia Severity Index; PSG, polysomnography; RMMA, rhythmic masticatory muscle activity (episodes per hour of sleep).

**FIGURE 2:**
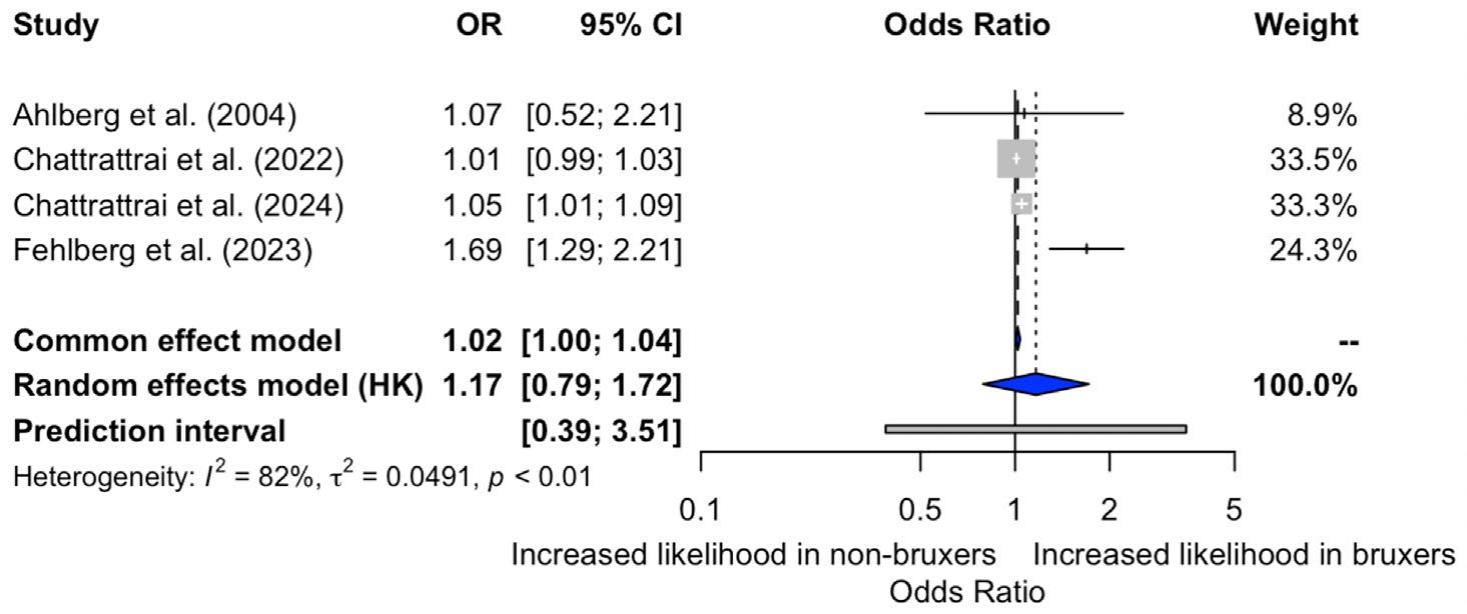
Meta‐analysis of the relationship between the presence of insomnia and self‐reported SB.

A meta‐analysis of four studies assessing the association between insomnia and self‐reported SB revealed a statistically significant association under the common‐effect model (OR 1.02; 95% CI 1.00–1.04), and a nonsignificant association under the random‐effects model (OR 1.17; 95% CI 0.79–1.72). Substantial heterogeneity was observed across studies (*I* ^2^ = 82%, *p* < 0.01), suggesting variability in the magnitude of the association (Figure 2).

**FIGURE 3:**
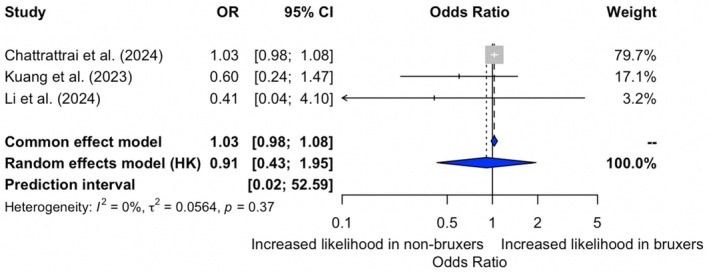
Meta‐analysis of the relationship between presence of insomnia and PSG‐based SB.

Figure 3 displays a meta‐analysis examining the association between insomnia and PSG‐based SB, which included three studies. Under the common‐effect model, the overall OR was 1.03 (95% CI: 0.98–1.08), indicating no statistically significant association. Similarly, the random‐effects model showed a nonsignificant association (OR 0.91; 95% CI 0.43–1.95). Heterogeneity across studies was low (*I* ^2^ = 0%, *p* = 0.37), suggesting consistency in the effect estimates.

The certainty of the evidence for both outcomes was present in Table 2. For the association between insomnia and self‐reported SB, the certainty was rated as very low, primarily due to serious inconsistency (*I* ^2^ = 82%), risk of bias, and serious imprecision, as the confidence interval included the null effect and the prediction interval was wide. The population, exposure, and outcome were judged to be directly applicable. The certainty of evidence for the association between insomnia and PSG‐based SB was rated as low. Although heterogeneity was low (*I* ^2^ = 0%) and the outcome measurement methods were appropriate, some studies had a risk of bias and small sample size, beyond which the confidence intervals were wide, reflecting substantial uncertainty in the estimate and serious imprecision.

**TABLE 2:** Analysis of certainty of evidence of studies included.

Insomnia and self‐reported sleep bruxism (*n* = 6)								
GRADE					Other considerations			
Study design	Risk of bias	Inconsistency	Indirectness	Imprecision	Participants	Effect (random) (OR, 95% CI)	Certainty of evidence	Importance
Observational	Serious	Serious	Not serious	Serious	~5500	1.17 (0.79–1.72)	Very low ⬤◯◯◯	Critical

Insomnia and PSG‐based sleep bruxism (*n* = 5)								
GRADE					Other considerations			
Study design	Risk of bias	Inconsistency	Indirectness	Imprecision	Participants	Effect (random) (OR, 95% CI)	Certainty of evidence	Importance
Observational	Serious	Not serious	Not serious	Serious	~1400	0.91 (0.43–1.95)	Low ⬤⬤◯◯	Critical

Abbreviations: CI, confidence interval; GRADE, Grading of Recommendations Assessment, Development and Evaluation (GRADE) approach; OR, odds ratio.

## 4 Discussion

This systematic review and meta‐analysis aimed to investigate the association between insomnia and SB, considering both self‐reported and PSG‐based assessments. Our findings suggest that there is no consistent evidence supporting a significant relationship between insomnia and SB. While the fixed‐effects model showed a statistically significant association between insomnia and self‐reported SB, this was not confirmed under the random‐effects model, nor was any association found for PSG‐based bruxism across models. These discrepancies highlight the heterogeneity among studies and the potential influence of methodological varieties in meta‐analytic outcomes.

Previous literature has suggested a plausible biological basis for the association between insomnia and SB, as both are influenced by CNS mechanisms, particularly those involving hyperarousal and stress‐related pathways [32, 33]. Insomnia is characterised by increased physiological and cognitive arousal, which may disrupt sleep continuity and trigger rhythmic masticatory muscle activity, a hallmark of SB [34, 35]. Moreover, both conditions share common psychosocial risk factors such as anxiety and emotional stress, which can exacerbate nocturnal motor activity [28, 33]. Despite this biological plausibility, the findings from the primary studies included in the systematic review are inconsistent. Some investigations observed a positive association between insomnia and self‐reported SB [26, 27], while others found no significant link [28, 31]. Similarly, studies using PSG to assess SB also reported no clear association with insomnia levels and this device‐based assessment [26, 29, 30].

These discrepancies may be attributed to differences in SB measurement methods, sample characteristics, and the presence of confounding factors across studies. While self‐reported measures are more accessible, they are prone to bias. Additionally, the methodological and statistical differences among the primary studies using self‐reported SB—such as varied assessment criteria and confounding adjustments—could have contributed to the heterogeneity and affected the pooled estimates. Importantly, the inconsistent definitions and assessment tools used to evaluate both insomnia and SB across studies further limit comparability and reduce confidence in the pooled results. Lastly, the sample sizes and precision of the included studies also influenced the weights attributed in the meta‐analysis models. The current evidence does not allow for a definitive conclusion and highlights the need for further research employing standardised assessment criteria, such as instrumental methods (e.g., PSG), in future studies investigating the association between insomnia and SB, as well as more representative samples.

In this study, we employed both random‐effects and fixed‐effects models to analyse the association under investigation. The random‐effects model did not reveal a statistically significant association. However, the fixed‐effects model indicated a significant association, suggesting a potential underlying trend. The decision to present results from the random‐effects model was made to account for potential variability across studies and to provide a more conservative estimate [36]. The significant heterogeneity observed (*I* ^2^ = 82%) in the self‐reported SB analysis supports the use of a random‐effects model. These findings emphasise the need for caution in interpreting pooled results when substantial heterogeneity is present. Nevertheless, despite the substantial heterogeneity observed, subgroup analyses or meta‐regression were not conducted due to the limited number of included studies, which would result in insufficient statistical power. Furthermore, while the random‐effects model did not yield a significant result, it approached significance, highlighting the possibility of an association that may become apparent with additional data or larger sample sizes.

This systematic review and meta‐analysis has some limitations that should be considered. The small number of eligible studies (four for self‐reported SB and three for PSG‐based SB) limited our statistical power and prevented meaningful subgroup analyses or meta‐regression to explore sources of heterogeneity. This constraint is particularly relevant given that half of the included studies showed a moderate risk of bias, potentially affecting the reliability of both individual findings and pooled estimates, while also reducing the overall certainty of evidence [37]. These methodological challenges are further compounded by the heterogeneity in how both insomnia and SB were defined and assessed across studies, especially for self‐reported measures, which contributed to the observed inconsistencies in our results and ultimately limited the strength of the review's findings. Although we conducted a comprehensive literature search, we were unable to formally assess publication bias due to the limited number of studies, though its potential influence cannot be discounted.

Given these limitations, our findings suggest that the current evidence base remains insufficient to draw definitive conclusions about the relationship between insomnia and SB. This conclusion emphasises the need for additional high‐quality studies that employ standardised assessment criteria for both conditions. Such future research should particularly focus on investigating potential moderating factors including age, sex, stress levels, and comorbid sleep disorders, as well as examining circadian patterns of bruxism, as these elements may help explain the conflicting findings present in the current literature. This highlights the need to phenotype our patients, allowing a better approach to specific groups.

The importance of this systematic inquiry becomes clear when considering the high population prevalence of both insomnia and SB [5, 6, 7, 38] and their likely shared pathophysiological mechanisms involving hyperarousal, stress dysregulation, and sleep fragmentation. It is important to emphasise that there is significant evidence of this association in specific clusters, such as middle‐aged women [16]. By systematically comparing findings across different SB assessment methods, our review not only identifies critical gaps in the current literature but also demonstrates how measurement approaches may influence observed associations between these conditions. While the limitations we identified temper the strength of our conclusions, this work nevertheless provides a necessary foundation for future research and a clearer understanding of the methodological challenges that must be addressed to advance our knowledge of the insomnia–SB relationship.

## 5 Conclusion

This systematic review and meta‐analysis found no consistent evidence supporting an association between insomnia and SB in adults. The reduced number and moderate risk of bias in some included studies, combined with the heterogeneity of definitions and assessment methods, limit the certainty of the evidence on this topic. This review emphasises the need for new studies with sufficient power to better understand the putative association between insomnia and SB. We emphasise the need to evaluate clusters within this possible association and phenotype patients, allowing for a better therapeutic approach.

## Author Contributions

Study design was carried out by all authors. Study selection and data extraction were conducted by E.M. and J.K.K. Meta‐analysis was conducted by E.M., J.K.K., and F.B.Z. Writing was conducted by E.M. and J.K.K. Critical review was performed by E.M., J.K.K., F.B.Z., C.D.F., and D.P.

## Conflicts of Interest

The authors declare no conflicts of interest.

## Supporting information


**Table S1:** Eletronic databases searched and search strategies used in the systematic review (up to March 2025).

## Data Availability

The data that support the findings of this study are available from the corresponding author upon reasonable request.

## References

[1] American Academy of Sleep Medicine (AASM) , International Classification of Sleep Disorders: Diagnostic and Coding Manual, 3rd ed. (American Academy of Sleep Medicine, 2014).

[2] American Academy of Sleep Medicine (AASM) , International Classification of Sleep Disorders – Third Edition (ICSD‐3), 3rd ed., text revision (ICSD‐3‐TR) (AASM International Classification of Sleep Disorders, 2023).

[3] American Psychiatric Association , Diagnostic and Statistical Manual of Mental Disorders (DSM‐5), 5th ed. (American Psychiatric Publishing, 2013).

[4] Associação Brasileira do Sono , Consenso Brasileiro de Insônia – 2024 (ABS, 2024), https://absono.com.br/wp‐content/uploads/2024/07/30332‐Consenso‐Brasileiro‐de‐Insonia.pdf.

[5] M. M. Ohayon , “Epidemiology of Insomnia: What We Know and What We Still Need to Learn,” Sleep Medicine Reviews 6, no. 2 (2002): 97–111, 10.1053/smrv.2002.0186. 12531146

[6] C. M. Morin , M. LeBlanc , L. Bélanger , et al., “Prevalence of Insomnia and Its Treatment in Canada,” Canadian Journal of Psychiatry 56, no. 9 (2011): 540–548, 10.1177/070674371105600904. 21959029

[7] F. Lobbezoo , J. Ahlberg , K. G. Raphael , et al., “International Consensus on the Assessment of Bruxism: Report of a Work in Progress,” Journal of Oral Rehabilitation 45, no. 11 (2018): 837–844, 10.1111/joor.12663. 29926505 PMC6287494

[8] M. C. Verhoeff , F. Lobbezoo , J. Ahlberg , et al., “Updating the Bruxism Definitions: Report of an International Consensus Meeting,” Journal of Oral Rehabilitation 52 (2025): 1335–1342, 10.1111/joor.13985. 40312776 PMC12408978

[9] G. Melo , J. Duarte , P. Pauletto , et al., “Bruxism: An Umbrella Review of Systematic Reviews,” Journal of Oral Rehabilitation 46, no. 7 (2019): 666–690, 10.1111/joor.12801. 30993738

[10] M. C. Carra , N. Huynh , and G. J. Lavigne , “Sleep Bruxism: A Comprehensive Overview for the Dental Clinician Interested in Sleep Medicine,” Dental Clinics of North America 56, no. 2 (2012): 387–413, 10.1016/j.cden.2012.01.002. 22480810

[11] S. Orzeszek , H. Martynowicz , J. Smardz , et al., “Assessment of the Relationship Between Sleep Bruxism, Reported Pain and Headache, Selected Health Factors, and General Health Conditions Among Temporomandibular Disorder Patients: A Preliminary Report,” Dental and Medical Problems 62, no. 2 (2025): 393–399, 10.17219/dmp/192824. 40407145

[12] A. Keskinruzgar , A. Kalenderoglu , G. Yapici Yavuz , et al., “Investigation of Neurodegenerative and Inflammatory Processes in Sleep Bruxism,” Cranio 38, no. 6 (2020): 358–364, 10.1080/08869634.2018.1543829. 30406732

[13] M. Michalek‐Zrabkowska , M. Wieckiewicz , J. Smardz , et al., “Determination of Inflammatory Markers, Hormonal Disturbances, and Sleepiness Associated With Sleep Bruxism Among Adults,” Nature and Science of Sleep 12 (2020): 969–979, 10.2147/NSS.S268470. PMC7667147 33204200

[14] T. Kato , N. M. Thie , N. Huynh , et al., “Topical Review: Sleep Bruxism and the Role of Peripheral Sensory Influences,” Journal of Oral Rehabilitation 28, no. 11 (2001): 957–965, 10.1046/j.1365-2842.2001.00821.x. 14520766

[15] M. Maluly , M. L. Andersen , C. Dal Fabbro , et al., “Polysomnographic Study of the Prevalence of Sleep Bruxism in a Population Sample,” Journal of Dental Research 92, no. 7 Suppl (2013): 97S–103S, 10.1177/0022034513496257. 23690359

[16] M. Maluly , C. Dal Fabbro , M. L. Andersen , A. Herrero Babiloni , G. J. Lavigne , and S. Tufik , “Sleep Bruxism and Its Associations With Insomnia and OSA in the General Population of Sao Paulo,” Sleep Medicine 75 (2020): 141–148, 10.1016/j.sleep.2020.06.016. 32858352

[17] B. Blaszczyk , M. Meira E Cruz , M. Waliszewska‐Prosol , et al., “Sleep Bruxism and Sleep Structure in Comorbid Insomnia and Obstructive Sleep Apnea (COMISA) Syndrome: A Polysomnographic Study,” Journal of Clinical Medicine 13, no. 11 (2024): 3154, 10.3390/jcm13113154. 38892864 PMC11172901

[18] C. H. Bastien , A. Vallières , and C. M. Morin , “Validation of the Insomnia Severity Index as an Outcome Measure for Insomnia Research,” Sleep Medicine 2, no. 4 (2001): 297–307, 10.1016/s1389-9457(00)00065-4. 11438246

[19] G. A. Wells , The Newcastle‐Ottawa Scale (NOS) for Assessing the Quality if Nonrandomized Studies in Meta‐Analyses (Ottawa Hospital Research Institute, 2009).

[20] C. K. Lo , D. Mertz , and M. Loeb , “Newcastle‐Ottawa Scale: Comparing Reviewers' to Authors' Assessments,” BMC Medical Research Methodology 14, no. 1 (2014): 45, 10.1186/1471-2288-14-45. 24690082 PMC4021422

[21] G. H. Guyatt , A. D. Oxman , G. E. Vist , et al., “GRADE: An Emerging Consensus on Rating Quality of Evidence and Strength of Recommendations,” BMJ (Clinical Research Ed.) 336, no. 7650 (2008): 924–926, 10.1136/bmj.39489.470347. PMC2335261 18436948

[22] H. J. Schünemann , P. Tugwell , B. C. Reeves , et al., “Non‐Randomized Studies as a Source of Complementary, Sequential or Replacement Evidence for Randomized Controlled Trials in Systematic Reviews on the Effects of Interventions,” Research Synthesis Methods 4, no. 1 (2013): 49–62, 10.1002/jrsm.1078. 26053539

[23] R. L. Grant , “Converting an Odds Ratio to a Range of Plausible Relative Risks for Better Communication of Research Findings,” BMJ (Clinical Research Ed.) 348 (2014): f7450, 10.1136/bmj.f7450. 24464277

[24] D. B. Richardson , A. C. Kinlaw , R. F. MacLehose , and S. R. Cole , “Standardized Binomial Models for Risk or Prevalence Ratios and Differences,” International Journal of Epidemiology 44, no. 5 (2015): 1660–1672, 10.1093/ije/dyv137. 26228585 PMC6372130

[25] J. P. T. Higgins , J. Thomas , J. Chandler , et al., eds., Cochrane Handbook for Systematic Reviews of Interventions Version 6.5 (Updated August 2024) (Cochrane, 2024), www.cochrane.org/handbook.

[26] T. Chattrattrai , G. Aarab , T. F. Blanken , et al., “Network Analysis of Sleep Bruxism in the EPISONO Adult General Population,” Journal of Sleep Research 33, no. 2 (2024): e13957, 10.1111/jsr.13957. 37246335

[27] B. K. Fehlberg , M. B. A. Barros , and M. G. Lima , “Health Behaviors and Multimorbidity Associated With Bruxism: Population‐Based Study,” Oral Diseases 29, no. 1 (2023): 245–253, 10.1111/odi.13928. 34056810

[28] T. Chattrattrai , T. F. Blanken , F. Lobbezoo , N. Su , G. Aarab , and E. J. W. van Someren , “A Network Analysis of Self‐Reported Sleep Bruxism in The Netherlands Sleep Registry: Its Associations With Insomnia and Several Demographic, Psychological, and Life‐Style Factors,” Sleep Medicine 93 (2022): 63–70, 10.1016/j.sleep.2022.03.018. 35429746

[29] B. Kuang , G. Aarab , Y. Wei , et al., “Associations Between Signs of Sleep Bruxism and Insomnia: A Polysomnographic Study,” Journal of Sleep Research 32, no. 4 (2023): e13827, 10.1111/jsr.13827. 36703561 PMC10909425

[30] D. Li , F. Lobbezoo , A. A. J. Hilgevoord , N. de Vries , and G. Aarab , “Prevalence and Risk Factors of Sleep Bruxism in Adults With Primary Snoring: A Large‐Scale Polysomnographic Study,” Journal of Clinical Sleep Medicine 20, no. 8 (2024): 1331–1337, 10.5664/jcsm.11142. 38607243 PMC11294144

[31] J. Ahlberg , A. Savolainen , M. Rantala , H. Lindholm , and M. Könönen , “Reported Bruxism and Biopsychosocial Symptoms: A Longitudinal Study,” Community Dentistry and Oral Epidemiology 32, no. 4 (2004): 307–311, 10.1111/j.1600-0528.2004.00163.x. 15239782

[32] G. J. Lavigne , N. Huynh , T. Kato , et al., “Genesis of Sleep Bruxism: Motor and Autonomic‐Cardiac Interactions,” Archives of Oral Biology 52, no. 4 (2007): 381–384, 10.1016/j.archoralbio.2006.11.017. 17313939

[33] M. H. Bonnet and D. L. Arand , “Hyperarousal and Insomnia: State of the Science,” Sleep Medicine Reviews 14, no. 1 (2010): 9–15, 10.1016/j.smrv.2009.05.002. 19640748

[34] M. Wieckiewicz , A. Paradowska‐Stolarz , and W. Wieckiewicz , “Psychosocial Aspects of Bruxism: The Most Paramount Factor Influencing Teeth Grinding,” BioMed Research International 2014 (2014): 469187, 10.1155/2014/469187. 25101282 PMC4119714

[35] M. I. Fluerașu , I. C. Bocsan , S. Buduru , et al., “The Correlation Between Sleep Bruxism, Salivary Cortisol, and Psychological Status in Young, Caucasian Healthy Adults,” Cranio 39, no. 3 (2021): 218–224, 10.1080/08869634.2019.1619250. 31131730

[36] M. Borenstein , L. V. Hedges , J. P. T. Higgins , and H. R. Rothstein , Introduction to Meta‐Analysis (Wiley, 2009).

[37] B. J. Shea , B. C. Reeves , G. Wells , et al., “AMSTAR 2: A Critical Appraisal Tool for Systematic Reviews That Include Randomized or Non‐Randomized Studies of Healthcare Interventions, or Both,” BMJ (Clinical Research Ed.) 358 (2017): j4008, 10.1136/bmj.j4008. PMC5833365 28935701

[38] D. Manfredini , E. Winocur , L. Guarda‐Nardini , D. Paesani , and F. Lobbezoo , “Epidemiology of Bruxism in Adults: A Systematic Review of the Literature,” Journal of Orofacial Pain 27, no. 2 (2013): 99–110, 10.11607/jop.921. 23630682

