# Supplementary material for: Is Insomnia Linked to Sleep Bruxism in Adults? A Systematic Review and Meta‐Analysis
Source: J Oral Rehabil. 2025 Oct 4;53(1):257–64. doi: 10.1111/joor.70068 (PMC12705292; doi:10.1111/joor.70068)
Supplement: Supplementary file 1 — Table S1: Eletronic databases searched and search strategies used in the systematic review (up to March 2025). [file JOOR-53-257-s001.docx]

**Supplementary table 1.** Eletronic databases searched and search strategies used in the systematic review (up to March 2025).

| **Database** | **Search strategy used** | **Hits** |
| --- | --- | --- |
| Pubmed/MEDLINE  (https://www.ncbi.nlm.nih.gov/pubmed) | ((adults) OR (older adults)) OR (grown person))) AND ((((((chronic insomnia [MeSH Major Topic]) OR (insomnia)) OR (sleep disorders)) OR (difficulty sleeping) OR (micro-awakenings)) AND (((((("sleep bruxism"[MeSH Major Topic]) OR (sleep bruxism) OR (bruxism) OR (grinding teeth)) OR (clench teeth))) | 762 |
| Embase  (https://www.embase.com) | ('adults'/exp OR adults) AND ('insomnia'/exp OR insomnia) AND ('disorder'/exp OR disorder) AND ('sleep'/exp OR sleep) AND ('bruxism'/exp OR bruxism) | 191 |
| Scopus  (https://www.scopus.com) | TITLE-ABS-KEY ("adults)*" AND TITLE-ABS-KEY ("insomnia") AND TITLE-ABS-KEY ("sleep disorder” OR “bruxism”) | 149 |
| Web of Science  (https://login.webofknowledge.com) | (adults*AND TOPIC: ("insomnia*" OR "sleep desorders" AND TOPIC: (“sleep bruxism OR bruxism” | 22 |
| Lilacs  (lilacs.bvsalud.org) | ((adults) AND (insomnia) AND (sleep bruxism) | 11 |
| Total |  | 1,135 |
